# Computer evaluation of the EMIT assays carbamazepine, ethosuximide, phenobarbital, phenytoin, quinidine and theophylline on the Gemsaec centrifugal fast analyser

**DOI:** 10.1155/S1463924681000266

**Published:** 1981

**Authors:** Bengt Kinberger, Bengt-Åke Johansson

**Affiliations:** Department of Clinical Chemistry Central Hospital Halmstad 301 85 Sweden


the same principles. The instrument is easy to use once operators become familiar with its operation. Although the instrument was then evaluated for a few weeks no faults occurred which would raise doubts about its reliability in everyday use.

ACKNOWLEDGEMENTS
We thank the American Hospital Supply (U.K.) Ltd., for the loan of the instrument and the Department of Health for the financial support to enable us to carry out this evaluation. We are also grateful to Mr. J. Korten (Electronucleonics, Breda, Holland) and Mr. I. West (American Hospital Supply Company) for advice and assistance throughout the evaluation and to Mr. P. Brooks (Mayday Hospital, Croydon) for providing patient samples assayed for total protein.

REFERENCES

[1] Young, D. S. and Gochman, N. Standard methods of Clinical Chemistry, 1972, 7, 293.
[2] Henry, P., *Journal of Automatic Chemistry*, (1979), **1**, 195.
[3] Henry, P. and Saunders, R.A. *Annals of Clincial Biochemistry*, 1976, 13, 384.
[4] Savory, J., Heintges, M. G., Sonowane, M., and Cross, R. E. *Clinical Chemistry*, (1976), **22**, 1102.
[5] Blom, M. and Hjorne, N. *Clinical Chemistry*,(1975), **21**, 195.
[6] Fleetwood, J. A., Latner, A. L., Marriner, A., Skillen, A. W. and Smith, P. A. *Annals of Clinical Biochemistry*, (1977), **14**, 279.
[7] Ertingshausen, G., Amsellem-Winzelberg, L., Richert, J-F., and Davids, R. *Clinical Chemistry*, (1978), **24**, 1147.
[8] Brody, J. P., Valdes, R., Jnr. and Savory, J. *Clinical Chemistry*, (1979), **25**, 205.
[9] Cross, R. E., Heintges, M. G., Savory, J. and Wentz, P. W. *Clinical Chemistry*, (1976), **22**, 429.
[10] Van Stekelenburg, G. J., Valk, C., Van De Kamp, J. S., Van Wijngaarden-Penterman, M. J. G., and De Keijzer, M. H. *Clinica Chimica Acta*, (1978), **89**, 79.
[11] Fabiny, D. L., Ertingshausen, G., *Clinical Chemistry*, (1971), **17**, 696.
[12] Wentz, P. W., Cross, R. E. and Savory, J. *Clinical Chemistry*, (1976), **22**, 188.
[13] Cotlove, E., Harris, E. K. and Williams, G. Z. *Clinical Chemistry*, (1970), **16**, 1028.
[14] Tonks, D. B. *Clinical Chemistry*, (1963), 9, 217.
[15] Campbell, D. G. and Owen, J. A. *Australasian Annals of Medicine*, (1969), **18**, 4.
[16] Larsen, K. *Clinica Chimica Acta*, (1972), **41**, 209.
[17] Knoll, Von E., Rebel, F. C. and Wisser, H. *Journal of Clinical Chemistry and Clinical Biochemistry*, (1978), **16**, 239.
[18] Chasson, A., Grady, H. T., Stanley, M. A. *American Journal of Clinical Pathology*, (1961), **35**, 83.
[19] Gustaffsson, J. E. C., *Clinical Chemistry*, (1976), **22**, 616.
[20] Henry, P., and Saunders, R. A. *Annals of Clinical Biochemistry*, (1975), **12**, 119.
[21] Barnett, R. N. *The American Journal of Clinical Pathology*, (1968), **50**, 671.

### Computer evaluation of the EMIT assays carbamazepine, ethosuximide, phenobarbital, phenytoin, quinidine and theophylline on the Gemsaec centrifugal fast analyser

**Bengt Kinberger and Bengt-Åke Johansson**
*Department of Clinical Chemistry, Central Hospital, 301 85 Halmstad, Sweden*

### Introduction

The adaptation of EMIT antiepileptic drug assays to the Aminco Roto Chem II centrigufal fast analyser has been described by Finley et al [1,2]. The analysis procedure was very convenient and accurate; however, most of the data handling had to be done with the aid of a HP 9815A desktop computer. Using Finley's modification of the EMIT assays the authors wished to adapt EMIT similarly to the Gemsaec centrifugal fast analyser and perform all the data processing with the Gemsaec computer. As no EMIT-program was available it was decided to develop one that would operate for routine clinical chemistry work. The program was written in FOCAL 8 (the version used by Electro Nucleonics Inc., the manufacturer of the Gemsaec) computer language. The Gemsaec transfer disc has only 16 positions, so it was considered important not to occupy a large part of the disc with standards when analysing unknown samples.

### Apparatus

A Gemsaec centrifugal fast analyser attached to a PDP 8/e computer, with magnetic tape (dectape) as the storage device, was used for the evaluation. The Rotor temperature was kept within 37.0 ± 0.1°C. Electro Nucleonics Inc.'s loader for the Gemini analyser was used for the preparation of transfer discs for Gemsaec. The loader was prepared for the automatic delivering of two reagents and the sample flushed with buffer solution into the transfer disc.

### Reagents

Reagent kits from the Syva Corporation were used through out the evaluation.

*Stock solutions*: Reagents A, B, aed-buffer and calibration standards were reconstituted according to Syva's recommendations.

*Working solutions A and B* : Stock solutions A and B were diluted 10-fold with aed-buffer solution according to Finley et al (2).

*Flush solution* : aed-buffer solution.

### Instrumental parameters

*Gemsaec instructions* :
Reaction temperature : 37.0 ± 0.1°C
Wavelength : 340 nm
Filter position : 335-385 nm
Reaction mode : RATE
Initial reading : 45s
Reading interval : 60s
Number of readings : 5

*Computer instructions* :

| | | | |
|---|---|---|---|
| IR = 45 | TC = 17 ("address" to the EMIT program) |
| RI = 60 | CD = 1 |
| NR = 5 | AD = 4 |
| TF = 70 | SA = 1.2 |

### Transfer disc preparation

The transfer disc was prepared by means of totally automatic pipetting. 200 μl of working solution A was pipetted into well C of the disc. Simultaneously 200 μl of working solution B was pipetted into well B. 10 μl of the sample was flushed with 200 μl of aed-buffer solution into well C. The "sample holder" was loaded as follows: position 1 = deionised water, position 2 = zero calibration standard (blank), positions 3-7 = non-zero calibration standards (full set of standards) and positions 8-16 = unknown samples.

### Data handling

Five absorbance readings were taken on each of the rotor positions. The delta absorbance, i.e. the last reading minus the first reading, is a measure of the enzyme activity of the sample in its cuvet. The enzyme activity is correlated to the concentration of the drug in the sample. Similarly to Finley et al [1] the measured delta absorbances were treated as free counts of radioactivity in the radioimmunoassay. Applying the logit-log transformation used by Finley et al, in the computation of the standard curve, a second order, unweighted, non-linear curve provided a better fit for the experimental data than a linear curve did.

The following formulas were used as the basis of calculation of the FOCAL program developed here:

(1) $B = 1 - \Delta Abs$

$B0 = 1 - \Delta Abs_0$ $\quad \Delta Abs_0 = \Delta$ Abs of the blank

$Ra = (ratio) = B/B0$

$logit = \ln (Ra/(1-Ra)) \quad B < B0$

(2) $\ln(conc) = K \cdot logit^2 + L \cdot logit + M$

K,L and M factors were evaluated using the following matrix equation:

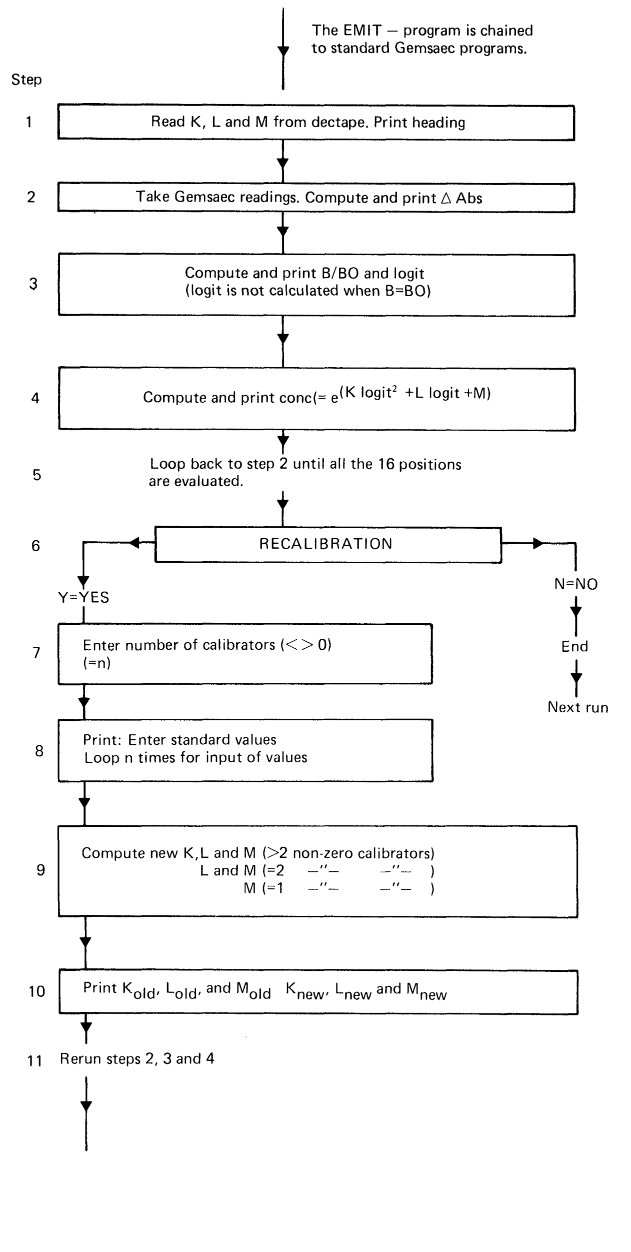

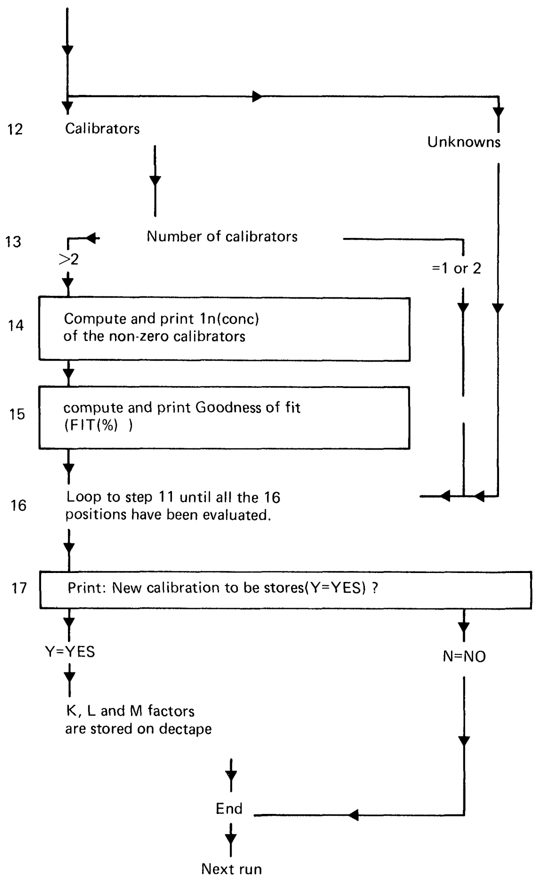

*Figure 1.*

$$(3)\quad \begin{bmatrix} \sum_{i=1}^{n} \text{logit}_i^2 & \sum_{i=1}^{n} \text{logit}_i & n \\ \sum_{i=1}^{n} \text{logit}_i^3 & \sum_{i=1}^{n} \text{logit}_i^2 & \sum_{i=1}^{n} \text{logit}_i \\ \sum_{i=1}^{n} \text{logit}_i^4 & \sum_{i=1}^{n} \text{logit}_i^3 & \sum_{i=1}^{n} \text{logit}_i^2 \end{bmatrix} * \begin{bmatrix} K \\ L \\ M \end{bmatrix} = \begin{bmatrix} \sum_{i=1}^{n} \ln(\text{conc})_i \\ \sum_{i=1}^{n} \text{logit}_i \cdot \ln(\text{conc})_i \\ \sum_{i=1}^{n} \text{logit}_i^2 \cdot \ln(\text{conc})_i \end{bmatrix}$$

(4) $\text{conc(unknown)} = e\ (K\cdot\text{logit}^2 + L\cdot\text{logit} + M)$

The curve fitting procedure was carefully inspected for falsely computed results from sera containing very low drug concentration. The logit-log parabola may reverse "direction" if B is close to BO.

K,L and M factors were stored on dectape in order to check the stability of the calibration between runs. During the first part of the program the calculation is carried out using the stored K,L and M factors in equation 4. If there is an unacceptable deviation from target values, e.g. when only standards are analysed, the curve fitting factors have to be recalculated. Three different modes of recalibration may be performed.

**Table 1. Complete calibration using five standards on each disc.**

| Assay | Sample | Target range/ value µmol/1 | Found: Mean µmol/1 | Found: Stand. dev. µmol/1 | Found: CV(%) |
|---|---|---|---|---|---|
| Carbamazepine | Seronorm* Pharmaca | 63 | 59.8(n=36) | 2.67 | 4.5 |
| | Syva's aed-control | 25.4 | 25.0(n=52) | 0.83 | 3.3 |
| | unknown | – | 4.1(n=7) | 0.30 | 7.3 |
| Ethosuximide | Syva's aed-control | 532 | 558.6(n=18) | 18.5 | 3.3 |
| | Seronorm Pharmaca diluted 1:2 | 210 | 215.5(n=36) | 10.8 | 5.0 |
| Phenobarbital | Syva's aed-control | 129.2 | 128.9(n=42) | 4.74 | 3.7 |
| | unknown | – | 107.7(n=9 ) | 4.20 | 3.9 |
| | Autonorm 50* | 60-72 | 75.1(n=35) | 0.97 | 1.3 |
| Phenytoin | Ortho III | 83 | 82.6(n=28) | 3.16 | 3.8 |
| | Syva's aed-control | 60 | 65.6(n=30) | 3.67 | 5.6 |
| | unknown | – | 7.3(n=6 ) | 0.77 | 10.5 |
| Quinidine | unknown | – | 24.2(n=18) | 0.46 | 1.9 |
| | Autonorm 50 | 8.5-13.7 | 13.5(n=27) | 0.43 | 3.2 |
| | Autonorm 50 diluted 1:4 | 2.1-3.6 | 3.3(n=36) | 0.16 | 4.8 |
| Theophylline | Ortho III | 111 | 99.3(n=35) | 3.36 | 3.4 |
| | unknown | – | 62.5(n=25) | 0.80 | 1.3 |
| | Ortho III diluted 1:5 | 22 | 19.0(n=18) | 0.74 | 3.9 |

*Manufactured by Nyegaard & Co., Oslo, Norway.

(A) *Recalibration using one standard in position 3 on each new disc.* (Pos 1=water and pos 2=blank). If a parallel shift of the standard curve has occurred in a new run, the following equations may be used for calculation:

(5) $\ln(\text{conc}) = K_{stored}\cdot\text{logit}^2 + L_{stored}\cdot\text{logit} + M_{new}$

The parallel shift may be corrected using the measured data of the standard positioned in cuvet number 3 of the rotor:

(6) $P3 = \ln(\text{conc})_3 - K\cdot\text{logit}_3^2$ index number refers to the position of the rotor

(7) $M_{new} = P3 - L_{stored}\cdot\text{logit}_3$

```
*G

CUV.  DELTA ABS.     B/B0        LOGIT      UMOL/L
 2     0.3186       1.0000
 3     0.6211       0.5561      0.2251      54.5
 4     0.5414       0.6730      0.7217      23.5
 5     0.5339       0.6840      0.7722      21.5
 6     0.5224       0.7009      0.8515      18.6
 7     0.5379       0.6781      0.7446      22.5
 8     0.4774       0.7669      1.1910      10.0
 9     0.3579       0.9423      2.7931       0.3
10     0.4664       0.7831      1.2836       8.4
11     0.4672       0.7819      1.2765       8.5
12     0.5364       0.6803      0.7548      22.1
13     0.5024       0.7303      0.9959      14.3
14     0.5717       0.6285      0.5253      33.0
15     0.4876       0.7520      1.1090      11.6
16     0.4954       0.7405      1.0485      13.0

RECALIBRATION (Y=YES)? Y

ENTER NUMBER OF STANDARDS : 2

ENTER STANDARD VALUES

 CUV.   3: 84.0

 CUV.   4: 33.6

 OLD CURVE:
 K= -    0.135
 L= -    1.568
 M=      4.358

 NEW CURVE:

 K= -    0.135

 L= -    1.717

 M=      4.824

CUV.  DELTA ABS.     B/B0        LOGIT      UMOL/L
 2     0.3186       1.0000
 3     0.6211       0.5561      0.2251      84.0
 4     0.5414       0.6730      0.7217      33.6
 5     0.5339       0.6840      0.7722      30.5
 6     0.5224       0.7009      0.8515      26.1
 7     0.5379       0.6781      0.7446      32.1
 8     0.4774       0.7669      1.1910      13.3
 9     0.3579       0.9423      2.7931       0.4
10     0.4664       0.7831      1.2836      11.0
11     0.4672       0.7819      1.2765      11.2
12     0.5364       0.6803      0.7548      31.5
13     0.5024       0.7303      0.9959      19.7
14     0.5717       0.6285      0.5253      48.6
15     0.4876       0.7520      1.1090      15.7
16     0.4954       0.7405      1.0485      17.7

 NEW CALIBRATION TO BE STORED (Y=YES)? N

DATA REVIEW?
```

*Figure 2. An outprint produced during the performance of a carbamazepine assay. Two standards, two quality controls and ten unknown samples were analysed. CUV 3 = 84 µmol/l: CUV 4 = 33.6 µmol/l; CUV 5 = Ortho III control serum, target value = 33.6 µmol/l; CUV 6 = Syva aed-control, target value = 25.4 µmol/l. The first part of the outprint shows that the stored calibration factors have to be corrected. The correction is shown in the second part.*

(B) *Recalibration using two standards in positions 3 and 4 on each new disc.*

The measured data of two standards positioned in cuvets 3 and 4 may be used for recalculation of two of the curve fitting factors K, L and M. We chose L and M factors for correction, as a correction of the K factor gave erroneous results:

(8) $P4 = \ln(\text{conc})_4 - K_{\text{stored}} \cdot \text{logit}_4^2$

(9) $P3 = \ln(\text{conc})_3 - K_{\text{stored}} \cdot \text{logit}_3^2$

(10) $P4 = L_{\text{new}} \cdot \text{logit}_4 + M_{\text{new}}$

(11) $P3 = L_{\text{new}} \cdot \text{logit}_3 + M_{\text{new}}$

(12) $L_{\text{new}} = (P4 - P3)/(\text{logit}_4 - \text{logit}_3)$

(13) $M_{\text{new}} = P4 - L_{\text{new}} \cdot \text{logit}_4 = P3 - L_{\text{new}} \cdot \text{logit}_3$

(C) *Recalibration using three or more standards on each new disc.*

This mode of calibration involves a new computation of all the three curve fitting factors K,L and M, according to formula 3. The calculated dose of each standard point is found using equation 4. Deviations from target values (deviations are expressed in the program as "Goodness of fit" in %) are calculated in percentages of the target values:

Calculated dose = $D_{\text{calc}}$

Actual dose (target value)= $D_{\text{act}}$

(14) Goodness of fit (%) = $100 \cdot (D_{\text{calc}} - D_{\text{act}})/D_{\text{act}}$

The flow chart of the computer program used is outlined in Figure 1.

### Results and discussion

The pipetting station, the loader, was carefully optimised before setting up EMIT runs. It was extremely important to prime the pump syringes prior to analysis. Table 1 shows the precision data obtained when five standards on each disc were used to calculate the standard curve. It is apparent that the coefficients of variation for the EMIT assays studied in this work were low. Unfortunately only nine positions were then left free on the disc for the evaluation of unknown samples. As the first section of the program makes no correction of the stored curve fitting factors, it was possible to check the agreement of standard curves between runs. However, results obtained from several runs of standard curves showed that the agreement was poor. Either a correction of the stored "standard curve" or a recalibration using a full set of standards (= five standards) must be performed. As stated in the data handling section, the facilities to correct a stored "standard curve" using measured data of either one or two standards on the disc were included in the program. Using just one standard for recalculation, the parallel shift correction, resulted in unreliable performance of the assay (results are not shown in this paper). However, recalibration using two standards was acceptable, see Table 2. Using this procedure an additional three samples per disc could be analysed, providing a more economical analysis than that performed with a full set of standards on each disc, although the latter was always superior to other modes of calibration. Two long-term quality control studies were made, see Table 3; they demonstrate the routine applicability and reliability of our computerised EMIT procedure.

Figures 2 and 3 show outprints produced during the execution of our program.

```
*G

 CUV.  DELTA ABS.     B/B0         LOGIT       UMOL/L
   2     0.3169      1.0000
   3     0.6652      0.4900 -     0.0400        83.1
   4     0.6099      0.5711       0.2861        49.3
   5     0.5731      0.6250       0.5104        33.9
   6     0.5152      0.7097       0.8937        17.3
   7     0.4649      0.7833       1.2846         8.3
   8     0.5377      0.6768       0.7387        22.8
   9     0.5907      0.5991       0.4015        40.7
  10     0.5372      0.6775       0.7419        22.7
  11     0.5282      0.6906       0.8027        20.3
  12     0.5356      0.6799       0.7529        22.2
  13     0.5334      0.6831       0.7678        21.6
  14     0.4881      0.7494       1.0951        11.9
  15     0.5507      0.6576       0.6526        26.5
  16     0.5585      0.6463       0.6026        28.9

 RECALIBRATION (Y=YES)? Y

ENTER NUMBER OF STANDARDS : 5

ENTER STANDARD VALUES

 CUV.    3: 84.0

 CUV.    4: 50.4

 CUV.    5: 33.6

 CUV.    6: 16.8

 CUV.    7: 8.4

 OLD CURVE:
 K= -    0.135
 L= -    1.568
 M=      4.358

 NEW CURVE:
 K= -    0.091
 L= -    1.638
 M=      4.375

 CUV.  DELTA ABS.     B/B0         LOGIT       UMOL/L    LN(C)      FIT(%)
   2     0.3169      1.0000
   3     0.6652      0.4900 -     0.0400        84.8    4.4306      1%
   4     0.6099      0.5711       0.2861        49.4    3.9197 -    2%
   5     0.5731      0.6250       0.5104        33.6    3.5142      0%
   6     0.5152      0.7097       0.8937        17.1    2.8211      2%
   7     0.4649      0.7833       1.2846         8.3    2.1279 -    1%
   8     0.5377      0.6768       0.7387        22.5
   9     0.5907      0.5991       0.4015        40.6
  10     0.5372      0.6775       0.7419        22.4
  11     0.5282      0.6906       0.8027        20.1
  12     0.5356      0.6799       0.7529        22.0
  13     0.5334      0.6831       0.7678        21.4
  14     0.4881      0.7494       1.0951        11.8
  15     0.5507      0.6576       0.6526        26.2
  16     0.5585      0.6463       0.6026        28.6

 NEW CALIBRATION TO BE STORED (Y=YES)? Y

  STANDARD CURVE STORED WITH HEADER  950*G

```

*Figure 3. A carbamazepine assay performed with a "full set" of standards on the disc. In this case the stored calibration factors are acceptable. However, the standard curve was recalibrated and the outprint from the curve fitting procedure is shown in the second part.*

### Conclusions

The EMIT assays of carbamazepine, ethosuximide, phenobarbital, phenytoin, quinidine and theophylline from the Syva Corporation are well suited for the Gemsaec centrifugal fast analyser. Results obtained show that the precision of the data fulfills the criteria for routine clinical chemistry.

By use of the specially designed calculation program for the Gemsaec computer, the manual plotting and evaluation is totally eliminated. No additional computer is required to perform calculation of EMIT results. Standard curves can be stored on magnetic tape and may be corrected by use of measured data of two standards in new runs. Three more samples per disc could therefore be analysed.

A great advantage of the EMIT assays is the enormous time saving as compared to other methods of analysis (GC or HPLC). EMIT enhances the possibility of producing fast reports both during the night and the day for the clinician. In an emergency situation this is a great asset for guiding the treatment of drug-intoxicated patients or underdosed patients.

REFERENCES

[1] Finley P. R., Williams R. J., Lichti D. F. and Byers III J. M., Assay of Phenobarbital: Adaptation of "EMIT" to the Centrifugal Analyser, *Clinical Chemistry* 23, 4, 738-740 (1977)

[2] Finley P. R., Williams R. J. and Byers III J. M., Assay of Phenytoin: Adaptation of "EMIT" to the Centrifugal Analyser, *Clinical Chemistry* 22, 6, 911-914 (1976)

**Table 2. Recalculation of the stored curve fitting factors L and M (see equation 2) of a "good" standard curve (ΣAbs (FIT (%) )=5). Number of standards on each disc = 2**

Assay: Theophylline

| | Standards | | "Unknowns" | | |
|---|---|---|---|---|---|
| Stated values μmol/1→ | 222 | 55.5 | 111 | 27.8 | 13.9 |
| Run | Found* | | Found | | |
| 1 | 222 | 55.5 | 108.9 | 28.0 | 14.4 |
| 2 | 222 | 55.5 | 108.9 | 26.9 | 12.1 |
| 3 | 222 | 55.5 | 114.5 | 26.1 | 13.3 |
| 4 | 222 | 55.5 | 117.1 | 26.3 | 12.0 |
| 5 | 222 | 55.5 | 116.2 | 26.3 | 12.7 |
| 6 | 222 | 55.5 | 115.4 | 28.6 | 13.6 |
| 7 | 222 | 55.5 | 113.3 | 27.3 | 13.6 |
| 8 | 222 | 55.5 | 108.6 | 27.4 | 14.1 |
| 9 | 222 | 55.5 | 113.8 | 27.4 | 13.8 |
| 10 | 222 | 55.5 | 117.7 | 26.6 | 13.9 |
| | | Mean = | 112.8 | 27.1 | 13.4 |
| | | Stand. dev. = | 3.17 | 0.81 | 0.82 |
| | | CV (%) = | 2.8 | 3.0 | 6.2 |

* *The values of the two standards used for calibration will always equal stated values.*

**Table 3. Long-term quality control**

| Assay | Period | Control | No. of obs. | Target μmol/1 | Found: Mean μmol/1 | Found: Stand. dev. μmol/1 | Found: CV(%) |
|---|---|---|---|---|---|---|---|
| Carbamazepine | June 1978-April 1980 | Syva's aed-control | 159 | 25.4 | 25.0 | 1.21 | 4.8 |
| | Sept 1979-April 1980 | Ortho III | 86 | 33.6 | 33.0 | 1.71 | 5.2 |
| Theophylline | June 1979-April 1980 | Syva's theophylline control | 75 | 83.0 | 82.3 | 2.69 | 3.3 |
| | June 1979-April 1980 | Ortho III | 73 | 111 | 105.1 | 4.10 | 3.9 |

